# Notes and News

**Published:** 1906-01-13

**Authors:** 


### Notes and News.

The Maharaja of Darbhanga, having given to the Prince of Wales a nazar, or present, of one lac of rupees, to be devoted to charitable objects, in commemoration of the Royal visit to India, the Prince has handed £6,000 to the Medical College at Calcutta.

A Bill which aims at the total prohibition of the manufacture of white lead has been prepared in France for introduction into the Chamber of Deputies. The Bill is the outcome of a recent Congress which assembled to consider the evils resulting from lead poisoning among painters. A clause has been inserted which will give the manufacturers concerned a respite of four years in which to adapt themselves to the new conditions, provided the Bill is passed.

At the quarterly meeting of the board of delegates of the Hospital Saturday Fund, held last week, the Council reported that the work of each department of the fund has shown an increase in the past year. They expressed their approval of the efforts which are being made by King Edward's Hospital Fund to bring about an amalgamation of the small special hospitals, and regard this movement as a step in the right direction, which should meet with the hearty co-operation of the managers of the institutions concerned.

The news reached Liverpool this week of the terrible fate of Mr. Stewart, of the Southern Nigerian Government. Dr. Stewart, who was riding a bicycle, started from Calabar River, and, getting ahead of his carriers, missed the main road and found himself in the village of a hostile tribe. He was never seen alive again, but the carriers came upon his bicycle lying in the trail, and making further search discovered portions of a human body which they identified as that of their late master. Their report showed that the body had not only been mutilated, but had been partially eaten by the murderers.

Dr. Osler, who is now in Ballarat, has authoritatively contradicted the report that he proposes to resign the Regius Professorship of Medicine in Oxford University.

A scheme has been established in London, the object of which is to secure for blind people who cannot afford to buy tickets free admission to musical and other entertainments. In order to be eligible to receive tickets, the blind must be personally known to a representative of the committee, so that their tastes and characters may be known. The public are asked to help in the work by giving subscriptions, and by sending spare tickets for any entertainments which are suitable. It is said that similar institutions recently started in the United States have proved very successful.

At an ordinary meeting of the Metropolitan Asylums Board, which took place last Saturday, the Ambulance Committee reported that their experience of motor-omnibuses had confirmed them in the opinion previously expressed, as to the suitability of motor traction for the purposes of the Board's ambulance service. The patients, they point out, would be in the vehicles for much shorter times, and consequently would suffer far less fatigue than in horse-drawn vehicles, and, as much more work could be performed in a day by motors than by horses, the committee believe that the general adoption of motor traction would result in considerable economy. They recommended the provision of an experimental motor establishment on a small scale at the south-western ambulance station. Their recommendations were adopted.

At a meeting held at Bristol in the Hall of the Society of Merchant Venturers last week, in aid of a scheme to raise £50,000 for the Bristol Royal Infirmary, the treasurer of the hospital, Sir George White, made the satisfactory announcement that of the £50,000 required more than £38,000 had been subscribed already. In the course of his address he quoted some interesting figures, which showed that in the year 1814 the income of the hospital from annual subscriptions and the number of annual subscribers were as large as they are at the present time, although the population has increased five-fold or six-fold in the intervening period. Sir George White further stated that the Bristol Royal Infirmary had never been out of debt during the last 43 years. Now it is certain that various improvements have been urgently required for some years past, and have been postponed only on account of the wretched financial condition of the hospital; and is interesting to reflect upon the additional proof thus afforded of the utter falsity of the doctrine that an institution ought to be constantly in debt in order to secure the sympathy and support of the public. We anticipate that the sound business principles which Sir George White intends to observe in the future conduct of its affairs will be the foundation of a new and larger life for the Bristol Royal Infirmary.

